# Effects of Body Orientation Relative to Gravity on Vection in Children and Adults

**DOI:** 10.1177/2041669520939585

**Published:** 2020-07-08

**Authors:** Keisuke Oyamada, Musashi Ujita, Tomoko Imura, Nobu Shirai

**Affiliations:** Department of Psychology, Graduate School of Arts and Letters, Tohoku University; Department of Information Systems, Faculty of Information Culture, Niigata University of International and Information Studies; Department of Psychology, Faculty of Integrated Arts and Social Sciences, Japan Women’s University; Department of Psychology, Faculty of Humanities, Niigata University

**Keywords:** vection, body axis, gravitational axis, development

## Abstract

We investigated the effects of the interaction between the body and gravitational axes on vection (visually induced self-motion perception) in school-age children and adults. Experiment 1 was a pilot study of adults that was conducted to determine the appropriate experimental settings for the main experiment that included children and adults. The adult participants experienced vection in four different directions in the head-centered coordinate (forward, backward, upward, and downward) under two postural conditions: standing (in which the body and gravitational axes were consistent) and supine (in which the body orientation was orthogonally aligned to the gravitational axis). The adults reported more rapid and longer lasting vection when standing than when supine. In the main experiment (Experiment 2), we tested adults and school-age children under conditions similar to those of Experiment 1 and found that the reported vection was more rapid and longer lasting in children than in adults, whereas the reported vection tended to be more rapid and longer lasting under the standing condition than the supine condition for both age groups. Based on the similarities and differences between children and adults found in the present and previous vection studies, child-specific features of vection are discussed.

Although visual information provides some of the most important cues about the surrounding environment, the mechanisms for processing visual information sometimes result in false recognition. When observing a visual pattern that covers most of the visual field and moves uniformly, individuals generally perceive a subjective self-motion that is opposite the direction of the pattern movement, even when the body remains still (Brandt et al., 1973). In addition to the classic definition of vection, additional definitions have been adopted in recent decades. Multiple sensory organs contribute to the self-motion perception, including visual, vestibular, somatosensory, and proprioceptive systems (Palmisano et al., 2015). Funatsu et al. (2013) reported that vection was facilitated by active breaststroke arm and body movements congruent with the self-motion direction. Vection can be perceived when the observer is moving or not. Palmisano et al. (2015) suggested that vection could be defined broadly as the conscious subjective experience self-motion.

Previous research has shown that even young children experience vection. For example, one of the earliest studies investigating vection during childhood (Lepecq et al., 1995) showed that 7- to 11-year-old children experienced vection in response to computer-displayed visual stimuli in an experimental situation. More recently, Shirai et al. (2012, 2014, 2018) reported that school-age children tended to report significantly more rapid and stronger vection than did adults. Although these previous studies showed that the experience of vection seems to differ between school-age children and adults, the developmental process of vection from childhood to adulthood remains unknown. Thus, we investigated the effects of nonvisual information, such as vestibular inputs or higher cognitive factors associated with the relationship between the body and the environment (e.g., Guterman & Allison, 2019; Lepecq et al., 1995), on vection in school-age children and adults.

The effects of the relationship between the orientation of an observer’s body axis and that of the gravitational axis on vection have attracted the interest of researchers. Although a number of previous studies have indicated that differences in an observer’s body angle relative to the gravitational axis can result in different experiences of vection, the findings have been inconsistent. For example, Kano (1991) reported that the experience of vection could be modulated by different postures, such as sitting and supine postures. In that study, vection stimuli, composed of moving dots displayed on two monitors placed on both lateral sides of a participant’s head, were presented in four different directions (forward, backward, upward, and downward) relative to the observer’s head-centered coordinate in each of the two postures. Thus, vection stimuli mainly appeared on the peripheral areas of the right and left visual fields of the participant. The results indicated that the latency of vertical vection (upward and downward) relative to the observer’s head-centered coordinate was significantly shorter than that of horizontal vection (forward and backward) in the sitting posture (when the body and gravitational axes are parallel). In addition, the mean latency of vection tended to be shorter in the downward and backward directions than the upward and forward directions relative to the observer’s head-centered coordinate in the supine condition (when the body and gravitational axes are orthogonal). In addition, Kano (1991) indicated that the latency of vection was shorter in the supine condition than in the sitting condition. Thus, the results of Kano (1991) indicate that the latency of vection can be modulated by changes in the relationship between the body axis and the gravitational axis.

On the other hand, Tovee (1999) showed inconsistent results with Kano (1991). Tovee (1999) presented visual stimuli eliciting an experience of the participants’ forward motion in a tunnel-like space displayed on a head-mounted display (HMD). The participants observed the visual stimuli with upright (seated) or supine postures. The results indicated that the latency of vection was significantly shorter in the upright posture than the supine posture. These results are inconsistent with those of Kano (1991), which showed shorter latency in the supine condition than in the upright posture condition. Guterman et al. (2012) reported results similar to Tovee (1999). They used visual stimuli composed of random blue spheres on a black background and displayed them on a PC display mounted in front of the participant’s head in each posture. Stimuli were presented in two directions (forward and backward). The participants observed stimuli in upright (seated), supine, prone, and left side down postures. They reported that the upright sitting posture promotes stronger vection than lying-down postures such as supine posture in two directions of visual stimuli. The results of Guterman and Allison (2019) also supported those of Tovee (1999) and Guterman et al. (2012). They manipulated the observer’s body posture (standing upright, prone, supine, lying on the right side down, and lying on the left side down) and presented visual flow stimuli composed of 3D dots or pipe-structured objects oriented vertically or horizontally in the virtual space. These stimuli were presented on a PC display mounted in front of the participant’s head in each posture. The observers reported stronger vection in the upright posture with the spinal flow stimulus compared with other conditions.

Several studies have reported findings that differ from the aforementioned previous studies. Nakamura and Shimojo (1998) investigated the body-tilt effect on vection in an experiment that required participants to observe visual stimuli composed of a random-dot pattern moving either horizontally or vertically while lying on an experimental bed. Visual stimuli were presented on the center of the screen in front of the participant using a video projector. The bed could be rotated with the participant’s frontal axis, and thus, the participant’s body angle relative to the visual stimuli could be maintained at arbitrary angles, that is, 0° (vertical), 30°, 45°, and 60° to the visual stimuli. The results showed that vertical but not horizontal vection was affected by the participant’s body tilt. The vertical vection was relatively strong when the body axis and direction of vection approached perpendicular, while the strength of vertical vection decreased when the body axis and direction of vection approached parallel. These results suggest that the known effect of the observer’s postures on experienced vection (e.g., Guterman & Allison, 2019; Guterman et al., 2012; Kano, 1991; Tovee, 1999) may extensively vary with the direction of the visual stimuli motion and the observer’s postures. In addition, Mori and Seno (2017) reported that experiences of vection can be modulated by more complex relationships among body postures and the gravity axis. They used visual stimuli composed of random-dot patterns that represented the participant’s forward/backward movements and displayed the stimuli on an HMD. The participants maintained three body postures (sitting, standing, or supine) and two head orientations (looking up or looking forward). They reported that stronger vection was experienced under the looking-forward head orientation than under the looking-up head orientation, whereas differences in the participants’ body posture had no effect on vection. These results showing the significant effect of the observer’s head (but not body) orientation on vection are inconsistent with the results of the aforementioned previous studies reporting significant effects of body postures on vection. Hummel et al. (2016) also demonstrated that a postural difference (upright vs. supine) had no effect on the perception of heading direction from visual information (optic flow stimuli), while the postural difference had a significant effect on the perception of heading direction from vestibular stimulation (physical motions presented by a moving platform). Although Hummel et al. (2016) did not directly assess observers’ experiences of vection, their results seem to be inconsistent with previous studies investigating the relationship among body postures and the gravity axis on perception of self-motion.

These inconsistencies among previous studies might stem from differences in experimental settings (e.g., visual stimuli and experimental apparatus). For example, Kano (1991) used two monitors placed on the lateral sides of the participant’s head to display visual stimuli, and thus, the visual stimuli appeared mainly in the peripheral visual field, whereas some other studies (Guterman et al., 2012; Nakamura & Shimojo, 1998) displayed a visual stimulus in front of the participant’s head, and thus, the visual stimulus covered most of the central area of the participant’s visual field. Tovee (1999) and Mori and Seno (2017) used an HMD and presented immersive simulated visual spaces to present visual stimuli. Due to apparatus variation in the previous studies, details of visual stimuli (such as size, shape, position in visual field, and so on) have varied considerably. It should be noted that the both size of visual stimuli and the visual stimuli area in the visual field (Brandt et al., 1973; Dichgans & Brandt, 1978; Johansson, 1977; Seno et al., 2009) can influence the experience of vection.

Despite the inconsistencies among previous studies, the interaction between an observer’s body (or head) orientation and the gravitational axis appears to have significant effects on vection. Therefore, we investigated the effect of the interaction between the body axis and the gravitational axis on vection by directly comparing children and adults under the same experimental conditions. Previous developmental research implies that there are commonalities as well as differences in the vection experience between children and adults. For example, both children and adults commonly experience more rapid vection during contracting optic flow than during expanding optic flow (Shirai et al., 2018). However, children experience significantly more rapid and stronger vection than do adults (Shirai et al., 2012, 2014, 2018). Determining whether body orientation has similar effects on vection in children and adults will be important in expanding scientific knowledge about the development of vection.

In the current study, we examined the effect of body orientation relative to the gravitational axis on vection by following the experimental setting used by Kano (1991) with several modifications. The reason why we adopted an experimental setup similar to that used by Kano (1991) was that we believed it would be the most appropriate setup for testing children, as compared with the other alternatives described earlier. For instance, if we adopted a computer display placed just in front of the participant’s head (as done by Guterman & Allison, 2019; Guterman et al., 2012), it might appear that the display could fall off onto the participant’s face in the supine position, which could invoke a fearful response in a young participant. Two displays placed on the lateral sides of the participant’s head, as used by Kano (1991), seemed to be a better solution. It should be noted that it is very important to provide a relaxing and secure atmosphere for testing young children. In addition, because the use of HMDs on children is controversial (cf. Yamada-Rice et al., 2017), we decided not to use any HMDs in our experiment.

This study included two experiments assessing vection. Experiment 1 was a pilot study of adult observers based on Kano (1991), with several modifications of the experimental design and procedures, which was conducted to determine the appropriate experimental settings for the main experiment with both adults and children. Experiment 2 (the main experiment) investigated the effects of the relationship between the body and gravitational axes on vection in children and adults.

## Experiment 1

Experiment 1 was a replication of Kano (1991), with several exceptions that were intended to remove potential confounding factors from the procedure. For example, Kano (1991) employed different procedures for measuring the latency of vection in the seated (responding with a foot pedal) and supine (responding by shutting eyes) conditions. This type of procedural difference between the two conditions might have affected the results; that is, shutting the eyes might have been easier and quicker than responding with a foot pedal, causing the apparent latency of vection to tend to be shorter in the supine condition than in the seated condition. In addition, the two experimental conditions in Kano (1991), the seated and supine conditions, were distinct in ways other than just the relationship between the observer’s body and gravitational axes. For example, the participants engaged in different body positions, such as bent knees in the seated condition versus straight knees in the supine condition, which may have affected the results in unexpected ways. Thus, in the present study, these potential confounding factors were removed from the procedure to the greatest extent possible. For example, the same response procedure (pressing a mouse button) and same body position (participants observed visual stimuli with straight knees) were used in both the standing and supine conditions.

### Methods

#### Ethics Statement

This study was approved by the Ethics Committee for Psychological Research at Niigata University, and all experiments were performed in accordance with the principles of the Declaration of Helsinki.

#### Participants

The final sample for Experiment 1 consisted of data obtained from 20 adult participants who were divided into 2 experimental groups: standing (*N* = 10, 6 females, mean age: 21.14 years, *SD*: ± 1.06 years, range: 19.70–22.41 years) and supine (*N* = 10, 5 females, mean age: 20.37, *SD*: ± 0.94 years, range: 18.99–22.24 years). All participants had normal or corrected-to-normal vision, and none had a history of visual or vestibular disease. Participants with a mean latency (or duration and magnitude) shorter (or larger) than the mean latency of each experimental group and/or than the group mean ± 3 *SD* for any of the three measurements (latency, duration, or magnitude) were to be excluded from the analyses, but no participants met these criteria. Two of the adult observers who participated in the initial experiment were excluded from the final sample due to apparatus failure.

#### Apparatus

**Figure 1.**
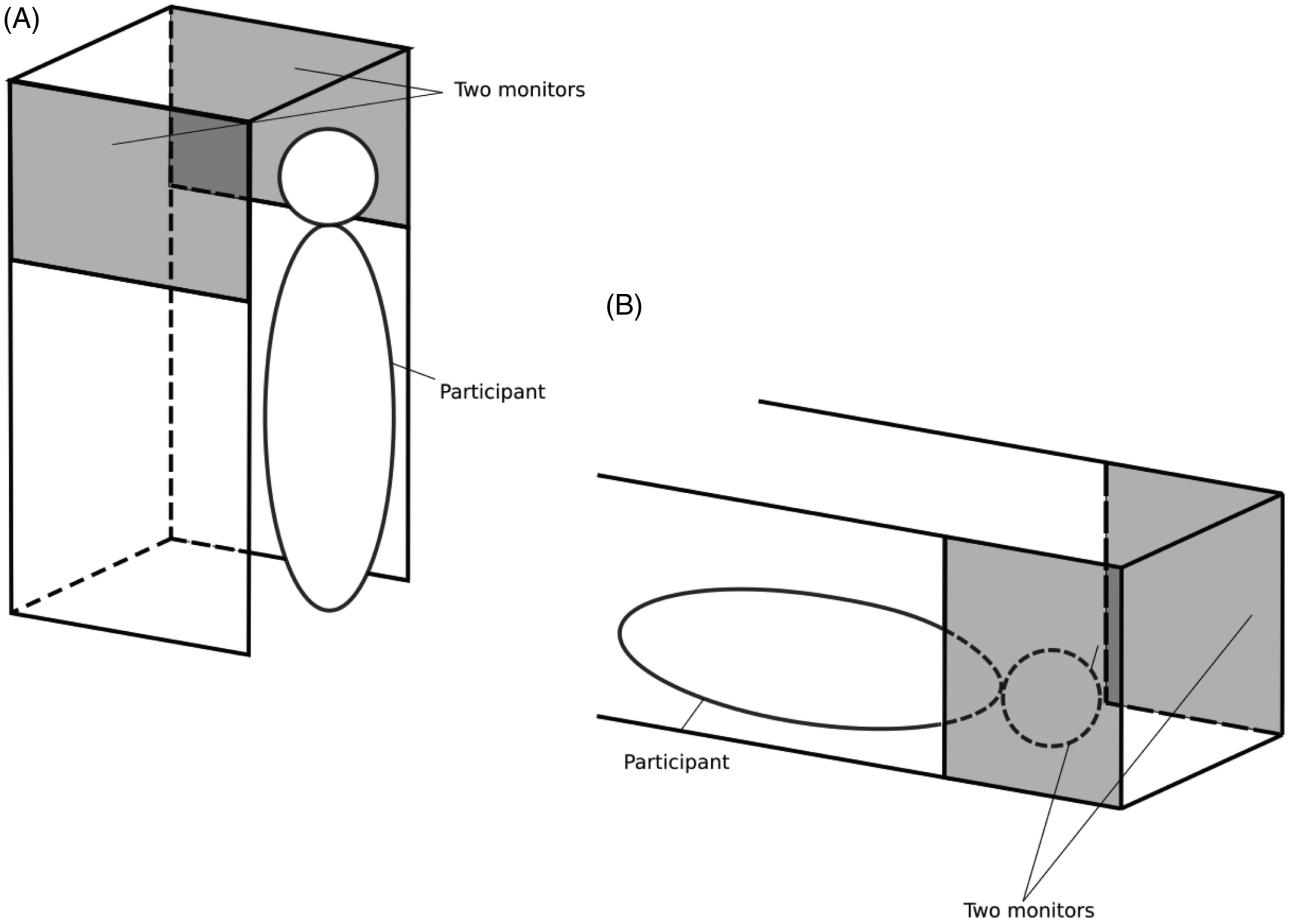
Schematic Illustration of the Experimental Chamber. (A) The chamber is used in the standing condition, and (B) the chamber is used in the supine condition. It should be noted that the chamber was fully covered with opaque black fabric during the experiments so that observers inside the chamber were not visible from outside the chamber under the experimental conditions.

**Figure 2.**
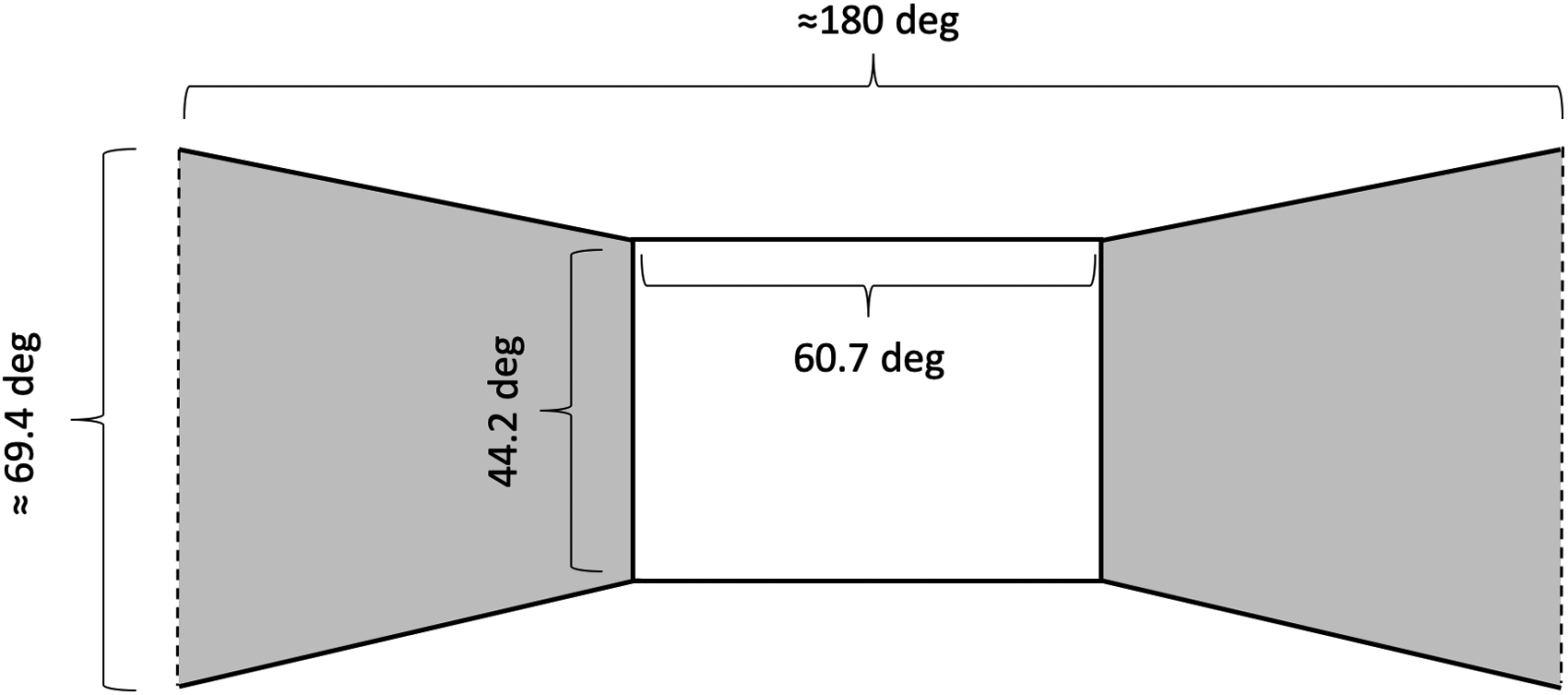
Schematic Illustration of a Subjective View of a Participant Inside the Experimental Chamber. Two gray trapezoids represent the two computer monitors displaying visual stimuli. The white rectangle represents a blank area in which no visual stimulus appeared. We calculated the visual angle of each side of the blank area by assuming that the typical viewing distance from the center of the blank area was 41.4 cm; subtracting a typical head length estimated as 18.4 cm from the width (59.8 cm) of each surface of the monitors (and thus, the length in depth of each visual stimulus). The estimated head length was calculated using a database of Japanese adult body sizes (Digital Human Laboratory, AIST, 2001). Because the database reported that the standard head length of young Japanese males and females was 18.91 cm and 17.85 cm, respectively, we decided to estimate the typical head length of our participants as the midpoint of these two reported values. The vertical visual angle of the most peripheral area of the visual stimuli (indicated by broken lines) was calculated by assuming that the whole horizontal angle of the visual scene was 180 degrees and that the viewing distance was 24.25 cm (the midpoint of the distance between the two computer monitors and thus, ideally corresponding to the center of the participant’s head). It should be noted that although our horizontal visual field typically subtends to more than 180 degrees under the binocular viewing condition, the size of the visual field potentially has individual differences. Thus, we adopted the convenient value of 180 degrees for calculation of the visual angles.

All experimental trials were conducted in a dimly illuminated room with an experimental chamber (59 cm wide × 178 cm high × 90 cm deep) that consisted of rigid iron frames covered with an opaque black fabric. In the standing condition, participants stood inside the experimental chamber and observed visual stimuli on two 27-inch LCD monitors (KA270HAbmidx, Acer Inc., New Taipei, Taiwan; Vsync: 60 Hz; resolution: 1,920 × 1,080 pixels; width: 59.8 cm; height: 33.6 cm) that were attached inside the experimental chamber (Figure 1). The two monitors were placed to the right and left sides of the participant, respectively, with their display surfaces facing toward the lateral sides of the participant’s head. The distance between the surfaces of the two monitors was 48.5 cm, and the heights of the monitors were adjustable so that the centers of the two monitors and the participant’s eyes could be aligned during the experimental sessions. A headrest was placed beside each participant’s neck to support the head during the experiments. In the supine condition, the entire chamber was placed sideways on the floor of the darkroom, and each participant observed the visual stimuli while lying on his/her back in the chamber (Figure 1). This setup gave each participant a viewing geometry as shown in Figure 2. It should be noted that the exact geometry was variable with the parameters of each participant’s head (e.g., head length and the distance between the eyes). Thus, the geometrical values shown in Figure 2 were calculated using standard parameters of head size of Japanese adults (Digital Human Laboratory, AIST, 2001).

The visual stimuli were generated and controlled by PsychoPy 2 software (Peirce et al., 2019) running on a personal computer (PC; LITTLEGEAR i310BA8, Mouse Computer Co., Ltd., Tokyo, Japan) that was located outside the experimental chamber. A computer mouse was connected to the PC and was used to retrieve the participants’ responses to the visual stimuli to measure latency and duration of vection.

#### Stimuli

Each visual stimulus was composed of 500 random white dots (diameter: 0.31 cm, 10 pixels) moving against a black background on each monitor. There were four conditions for the direction of the moving dots that corresponded to each participant’s head-centered coordinate: backward, forward, downward, and upward. Thus, the possible directions of induced vection in the head-centered coordinate based on the four directions of moving dots were forward, backward, upward, and downward. There were three speed conditions for the dots: low speed (4.44 cm/s), middle speed (6.52 cm/s), and high speed (17.44 cm/s). Each of the speed values corresponded to 10.5 deg/s, 15.4 deg/s, and 41.2 deg/s, respectively, from the midpoint between the surfaces of the two monitors (24.25 cm from each display). It should be noted that each of the speed conditions did not simulate a particular speed of self-motion in a geometrical space but simply replicated the speed conditions of Kano (1991). All dots moved at a constant speed (selected from one of the three speed conditions) in each experimental trial.

#### Procedure

All participants completed a total of 36 trials (4 directional conditions × 3 speed conditions × 3 repetitions) with a short break every 12 trials. Each participant was instructed to look at the frontal plane of the experimental booth and to try not to look at the LCD monitors during each trial. They were also instructed to hold the computer mouse in their dominant hand and to press the left button of the mouse when they experienced self-motion during each trial. After 30 s of stimulus presentation, each participant was asked to estimate the magnitude of self-motion during the stimulus presentation by drawing a short orthogonal line using a visual analogue scale (VAS; 100 mm in length). Each participant was instructed that if he/she felt no self-motion at all, then he/she should draw a vertical line at the left edge of the VAS. However, if he/she felt self-motion similar to real locomotion, then he/she should draw a line at the right edge of the VAS as a benchmark for the estimation. The distance from the left edge of the VAS to the intersection with the drawn line was adopted as the measurement of magnitude.

Two experimenters were involved in this procedure: the main experimenter, who selected an appropriate experimental trial and ran the relevant computer program to begin the experimental trial, and an assistant, who gave instructions to the participants and confirmed that each participant was looking at the front plane of the chamber and holding the computer mouse properly before the start of each trial. After each trial, the assistant gave the participant a pen and the VAS and confirmed that the participant marked the scale as instructed. The assistant was naïve to the properties of the visual stimuli in each trial.

### Results

#### Latency

**Figure 3.**
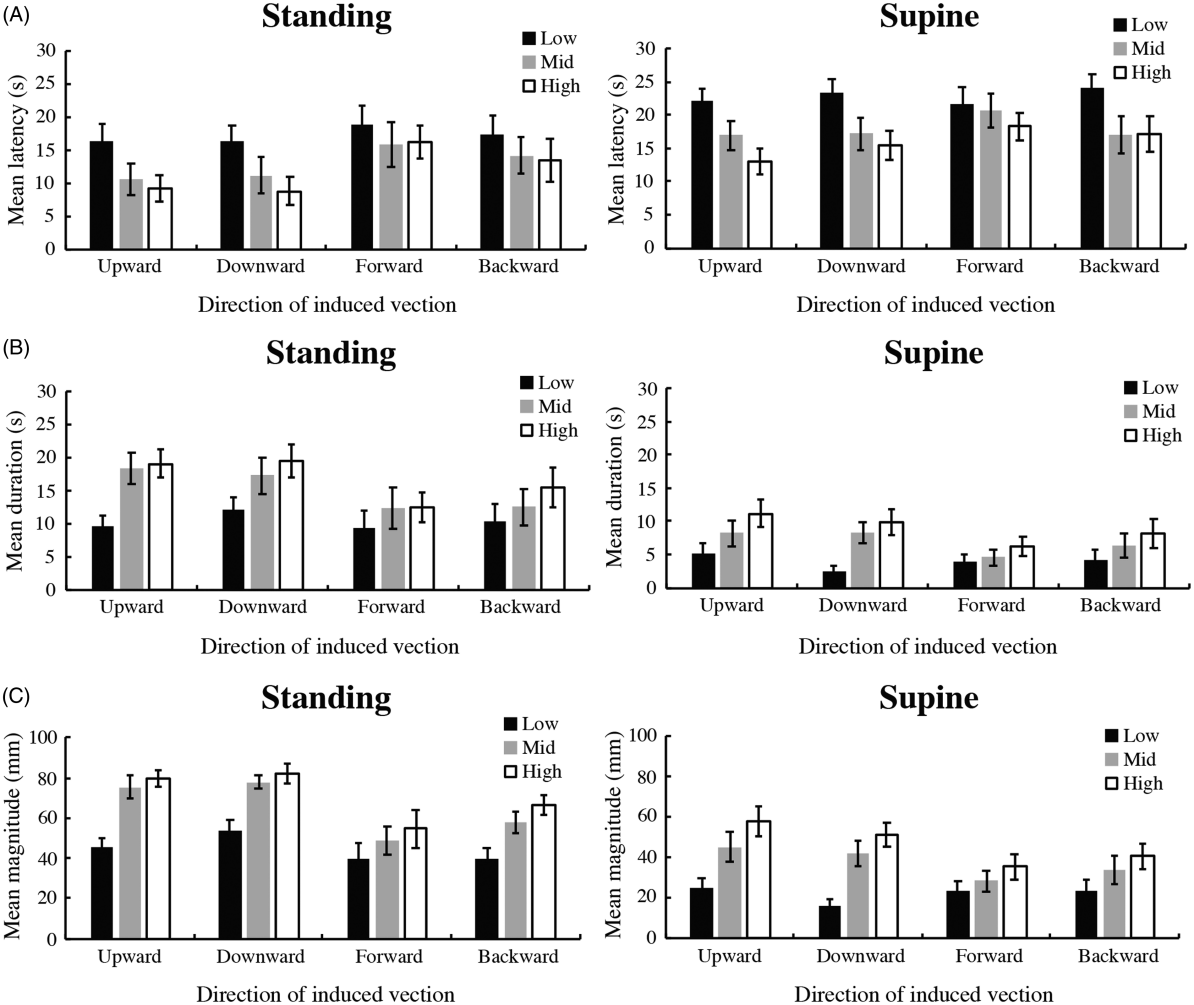
Latency (A), duration (B), and magnitude (C) measured in Experiment 1. The graphs on the left show results for the standing condition, whereas those on the right show results for the supine condition. In each graph, the black, gray, and white bars represent data in the low-, middle-, and high-speed conditions, respectively. It should be noted that the indicated directions (upward, downward, forward, and backward) represent the directions of vection in an observer’s head-centered coordinate. Error bars: ±1 *SEM*.

A three-way mixed analysis of variance (ANOVA; 2 posture × 4 directions of vection × 3 dot speed conditions) revealed that all three main effects were significant: posture, *F*(1, 18) = 5.814, *p* = .0268, ${\text{η}}_{\text{p}}^{2}$ = 0.244; direction, *F*(3, 54) = 3.657, *p* = .0179, ${\text{η}}_{\text{p}}^{2}$ = 0.169; and speed, *F*(2, 36) = 30.494, *p* < .0001, ${\text{η}}_{\text{p}}^{2}$ = 0.629 (Figure 3A). In addition, there was a significant interaction between direction and speed, *F*(6, 108) = 2.304, *p* = .0393, ${\text{η}}_{\text{p}}^{2}$ = 0.113. The simple main effects of direction of motion were significant in the middle, *F*(3, 162) = 3.474, *p* = .0175, ${\text{η}}_{\text{p}}^{2}$ = 0.060, and high, *F*(3, 162) = 5.665, *p* = .0010, ${\text{η}}_{\text{p}}^{2}$ = 0.095, speed conditions. For the middle-speed condition, multiple comparison analyses (two-tailed *t* tests with a Bonferroni correction) indicated that the mean latencies were shorter for the upward direction than for the forward direction and for the downward direction than for the forward direction (*p* values < .05/6). For the high-speed condition, multiple comparison analyses indicated that mean latencies were shorter for the upward direction than for the forward direction and for the downward direction than for the forward direction (*p* values < .05/6).

The simple main effects of speed on direction of vection were significant for upward, downward, and backward vections: upward, *F*(2, 144) = 18.831, *p* < .0001, ${\text{η}}_{\text{p}}^{2}$ = 0.207; downward, *F*(2, 144) = 16.117, *p* < .0001, ${\text{η}}_{\text{p}}^{2}$ = 0.183; and backward, *F*(2, 144) = 11.342, *p* < .0001, ${\text{η}}_{\text{p}}^{2}$ = 0.136. For each direction of vection, multiple comparison analyses indicated that mean latencies were shorter in the high-speed condition than in the low-speed condition and in the middle-speed condition than in the low-speed conditions (*p* values < .05/3). The other interactions did not reach significance: Posture × Direction, *F*(3, 54) = 0.355, *p* = .7856, ${\text{η}}_{\text{p}}^{2}$ = 0.019; Posture × Speed, *F*(2, 36) = 0.305, *p* = .7391, ${\text{η}}_{\text{p}}^{2}$ = 0.017; and Posture ×Direction × Speed: *F*(6, 108) = 0.972, *p* = .4479, ${\text{η}}_{\text{p}}^{2}$ = 0.051.

#### Duration

A three-way mixed ANOVA (2 posture × 2 direction of vection × 3 dot speed conditions) revealed that all three main effects were significant: posture, *F*(1, 18) = 14.305, *p* = .0014, ${\text{η}}_{\text{p}}^{2}$ = 0.443; direction, *F*(3, 54) = 4.971, *p* = .0041, ${\text{η}}_{\text{p}}^{2}$ = 0.216; and speed, *F*(2, 36) = 28.534, *p* < .0001, ${\text{η}}_{\text{p}}^{2}$ = 0.613 (Figure 3B). The interaction between direction and speed was also significant, *F*(6, 108) = 4.728, *p* = .0003, ${\text{η}}_{\text{p}}^{2}$ = 0.208. The simple main effects of direction of motion were significant for the middle, *F*(3, 162) = 6.347, *p* < .0001, ${\text{η}}_{\text{p}}^{2}$ = 0.105, and high, *F*(3, 162) = 8.293, *p* < .0001, ${\text{η}}_{\text{p}}^{2}$ = 0.133, speed conditions. For the middle-speed condition, multiple comparison analyses (two-tailed *t* tests with a Bonferroni correction) indicated that mean durations were longer for the upward direction than for the forward direction, for the upward direction than for the backward direction, and for the downward direction than for the forward direction (*p* values < .05/6). For the high-speed condition, multiple comparison analyses indicated that mean durations were longer for the upward direction than for the forward direction, for the upward direction than for the backward direction, for the downward direction than for the forward direction, and for the downward direction than for the backward direction (*p* values < .05/6).

The simple main effects of speed on direction of vection were significant for upward, downward, and backward vections: upward, *F*(2, 144) = 27.483, *p* < .0001, ${\text{η}}_{\text{p}}^{2}$ = 0.276; downward, *F*(2, 144) = 23.277, *p* < .0001, ${\text{η}}_{\text{p}}^{2}$ = 0.244; and backward, *F*(2, 144) = 7.690, *p* < .0007, ${\text{η}}_{\text{p}}^{2}$ = 0.096. For upward and downward vections, multiple comparison analyses indicated that mean durations were longer in the high-speed condition than in the low-speed condition and in the middle-speed condition than in the low-speed condition (*p* values < .05/3). For backward vection, multiple comparison analyses indicated that mean duration was longer in the high-speed condition than in the low-speed condition (*p* values < .05/3). The other interactions did not reach significance: Posture × Direction, *F*(3, 54) = 0.426, *p* = .7349, ${\text{η}}_{\text{p}}^{2}$ = 0.023; Posture × Speed: *F*(2, 36) = 1.150, *p* = .3279, ${\text{η}}_{\text{p}}^{2}$ = 0.060; and Posture ×Direction × Speed: *F*(6, 108) = 1.034, *p* = .4079, ${\text{η}}_{\text{p}}^{2}$ = 0.054.

#### Magnitude

A three-way mixed ANOVA (2 posture × 4 direction of vection × 3 dot speed conditions) revealed that all three main effects were significant: posture, *F*(1, 18) = 25.700, *p* < .0001, ${\text{η}}_{\text{p}}^{2}$ = 0.588; direction, *F*(3, 54) = 6.977, *p* = .0005, ${\text{η}}_{\text{p}}^{2}$ = 0.279; and speed, *F*(2, 36) = 60.041, *p* < .0001,  = 0.769 (Figure 3C). The interaction between direction and speed was also significant, *F*(6, 108) = 4.393, *p* = .0005, ${\text{η}}_{\text{p}}^{2}$ = 0.196. The simple main effects of direction of motion were significant in the middle, *F*(3, 162) = 7.963, *p* < .0001, ${\text{η}}_{\text{p}}^{2}$ = 0.129, and high, *F*(3, 162) = 10.113, *p* < .0001, ${\text{η}}_{\text{p}}^{2}$ = 0.158, speed conditions. In the middle- and high-speed conditions, multiple comparison analyses (two-tailed *t* tests with a Bonferroni correction) indicated that mean magnitudes were greater for the upward direction than for the forward direction, for the upward direction than for the backward direction, for the downward direction than for the forward direction, and for the downward direction than for the backward direction (*p* values < .05/6).

The simple main effects of speed on direction of vection were significant for all directions: upward, *F*(2, 144) = 39.606, *p* < .0001, ${\text{η}}_{\text{p}}^{2}$ = 0.355; downward, *F*(2, 144) = 30.778, *p* < .0001, ${\text{η}}_{\text{p}}^{2}$ = 0.299; forward, *F*(2, 144) = 3.535, *p* = 0.0317, ${\text{η}}_{\text{p}}^{2}$ = 0.047; and backward, *F*(2, 144) = 14.430, *p* < .0007, ${\text{η}}_{\text{p}}^{2}$ = 0.167. For the upward, downward, and backward vections, multiple comparison analyses indicated that the mean magnitudes were greater in the high-speed condition than in the low-speed condition and in the middle-speed condition then in the low-speed condition (*p* values < .05/3). For forward vection, multiple comparison analyses indicated that mean magnitude was greater in the high-speed condition than in the low-speed condition (*p* values < .05/3). The other interactions did not reach significance: Posture × Direction, *F*(3, 54) = 0.943, *p* = .4262, ${\text{η}}_{\text{p}}^{2}$ = 0.050; Posture × Speed: *F*(2, 36) = 0.590, *p* = .5596, ${\text{η}}_{\text{p}}^{2}$ = 0.032; and Posture × Direction × Speed: *F*(6, 108) = 0.516, *p* = .7955, ${\text{η}}_{\text{p}}^{2}$ = 0.028.

### Discussion

For all three measurements (latency, duration, and magnitude), there was a significant main effect of posture but no interaction effect. These results imply that the adult participants consistently reported more rapid and stronger vection in the standing condition than in the supine condition. In addition, for all three measurements, there was a significant interaction between direction and speed as well as significant results for the multiple comparison analyses of the simple main effects of vection direction in each speed condition. Taken together, these findings indicate that the participants tended to report more rapid and stronger vection for the vertical directions (upward and downward) than for the longitudinal directions (forward and backward) in the head-centered coordinate. Thus, two main conclusions were obtained from the results of Experiment 1. First, the adult participants reported more rapid and stronger vection in the standing condition in which the body axis was congruent with the gravitational axis compared with the supine condition in which the body and the gravitational axes were orthogonal. This finding is consistent with previous research reporting stronger vection in upright postures than in lying-down postures (Guterman & Allison, 2019; Guterman et al., 2012). Second, the participants showed an asymmetry between vection in the vertical directions and vection in the longitudinal directions; that is, they exhibited more rapid and stronger vection in the vertical directions than in the longitudinal directions in the head-centered coordinate but not the environment-centered coordinate.

The present findings are inconsistent with those of Kano (1991), who found that the latency of vection tended to be longer when the body axis was consistent with the gravitational axis (seated condition) than when the body axis was inconsistent with the gravitational axis (supine condition). Furthermore, although Kano (1991) reported an asymmetry between induced vection for the vertical and longitudinal directions in the seated condition, this asymmetry disappeared in the supine condition. That is, the asymmetry between vertical vection and longitudinal vection reported by Kano (1991) may have depended on the environment-centered but not the head-centered coordinate. The inconsistencies between the present and previous (Kano, 1991) findings may stem from differences in the experimental procedures. As discussed earlier, there were several potential confounding factors in the procedure of Kano (1991), but those factors were removed from the present experiment.

On the other hand, the results of Experiment 1 seem to be consistent with the previous studies that reported more rapid vection in upright postures than supine postures (Guterman & Allison, 2019; Guterman et al., 2012; Tovee, 1999). Thus, the present findings support the idea that vection is promoted more by the upright posture (in which the body axis is congruent with the gravity axis) than the supine or lying-down postures (in which the body axis is incongruent with the gravity axis), as shown in the previous studies. We discuss possible explanations for the promotion effect of vection in the upright posture in the General Discussion section.

## Experiment 2

The results of Experiment 1 indicated that adult observers experienced more rapid and stronger vection when the body and gravitational axes were congruent (standing condition) than when these axes were orthogonal (supine condition). Moreover, vection tended to be more rapid and stronger for the vertical directions than for the longitudinal directions in the head-centered coordinate. The aims of Experiment 2 were to investigate whether these two characteristics of vection in adults would be observed in younger individuals (6- to 11-year-old children) and to compare results directly between children and adults within an identical experimental setting.

### Methods

#### Participants

The final sample for Experiment 2 consisted of data obtained from 40 children and 40 adults who were each divided into two experimental groups (four total groups of participants): children in the standing condition (*N* = 20, 9 females, mean age: 9.20 years, *SD* = ± 1.81 years, range: 6.15–11.97 years), children in the supine condition (*N* = 20, 10 females, mean age: 8.83 years, *SD*: ± 1.59, range: 6.36–11.01 years), adults in the standing condition (*N* = 20, 10 females, mean age: 21.27 years, *SD*: ± 1.06, range: 19.22–22.73 years), and adults in the supine condition (*N* = 20, 11 females, mean age: 21.28 years, *SD*: ± 0.83, range: 19.74–22.59 years). All participants had normal or corrected-to-normal vision, and none had a history of visual or vestibular disease. The exclusion criteria applied in Experiment 1 were also applied in Experiment 2, but no participants met these criteria.

#### Apparatus, Stimuli, and Procedure

The apparatus, stimuli, and experimental procedures for Experiment 2 were essentially the same as those employed in Experiment 1, with the exception that only the high-speed condition (41.2°/s) was used. Because the younger children seemed to find it difficult to maintain motivation and concentration for a long period, it was necessary to shorten the number of trials as much as possible. The results of Experiment 1 indicated that the reported vection was most remarkable in the high-speed condition; thus, only the high-speed condition was adopted in Experiment 2 to shorten each set of experimental trials.

### Results

#### Latency

**Figure 4.**
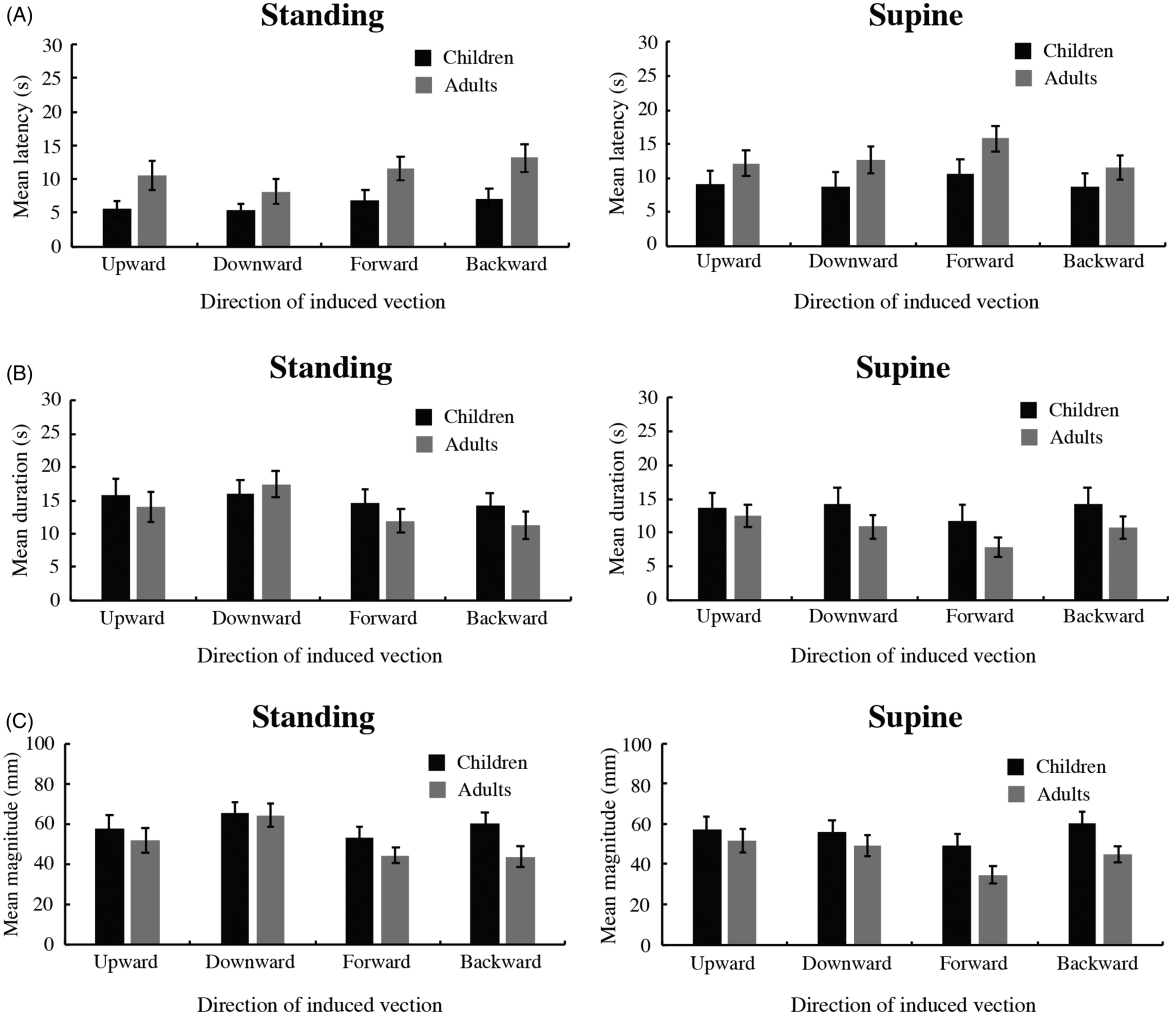
Latency (A), duration (B), and magnitude (C) measured in Experiment 2. The graphs on the left show results for the standing condition, and those on the right show results for the supine condition. In each graph, the black and gray bars represent the data of the children and adults, respectively. It should be noted that the indicated directions (upward, downward, forward, and backward) represent the directions of vection in an observer’s head-centered coordinate. Error bars: ±1 *SEM*.

A three-way mixed ANOVA (2 age × 2 posture × 4 direction conditions) revealed significant main effects of age, *F*(1, 76) = 7.158, *p* = .0091, ${\text{η}}_{\text{p}}^{2}$ = 0.086, and direction, *F*(3, 228) = 3.771, *p* = .0114, ${\text{η}}_{\text{p}}^{2}$ = 0.047. However, the main effect of posture was not significant, *F*(1, 76) = 2.826, *p* = .0969, ${\text{η}}_{\text{p}}^{2}$ = 0.036 (Figure 4A). There was also a significant interaction between posture and direction, *F*(3, 228) = 2.799, *p* = .0404, ${\text{η}}_{\text{p}}^{2}$ = 0.036. The simple main effects of posture were significant for downward vection, *F*(1, 304) = 4.679, *p* = .0313, ${\text{η}}_{\text{p}}^{2}$ = 0.015, and forward vection, *F*(1, 304) = 4.606, *p* = .0326,  = 0.015, indicating that latency was significantly shorter in the standing condition than in the supine condition when the visual stimuli represented downward and forward self-motion. The simple main effect of direction of vection on posture was significant for both the standing, *F*(3, 228) = 3.512, *p* < .0160, ${\text{η}}_{\text{p}}^{2}$ = 0.044, and supine, *F*(3, 228) = 3.058, *p* < .0291, ${\text{η}}_{\text{p}}^{2}$ = 0.039, conditions. Furthermore, for both the standing and supine conditions, multiple comparison analyses (two-tailed *t* tests with a Bonferroni correction) indicated that mean latency was shorter for downward vection than for backward vection (*p* values < .05/6). The other interactions did not reach significance: Age × Posture, *F*(1, 76) = 0.079, *p* = .7793, ${\text{η}}_{\text{p}}^{2}$ = 0.001; Age ×Direction, *F*(3, 228) = 0.377, *p* = .7699, ${\text{η}}_{\text{p}}^{2}$ = 0.005; and Age × Posture × Direction, *F*(3, 228) = 0.962, *p* = .4114, ${\text{η}}_{\text{p}}^{2}$ = 0.012.

#### Duration

A three-way mixed ANOVA (2 age × 2 posture × 4 direction conditions) revealed a significant main effect of direction, *F*(3, 228) = 7.662, *p* = .0001, ${\text{η}}_{\text{p}}^{2}$ = 0.092, whereas the main effects of age, *F*(1, 76) = 1.391, *p* = .2419, ${\text{η}}_{\text{p}}^{2}$ = 0.018, and posture, *F*(1, 76) = 1.683, *p* = .1984, ${\text{η}}_{\text{p}}^{2}$ = 0.022, did not reach significance (Figure 4B). There was also a significant interaction between posture and direction, *F*(3, 228) = 3.130, *p* = .0265, ${\text{η}}_{\text{p}}^{2}$ = 0.040. The simple main effect of posture was significant for downward vection, *F*(1, 304) = 4.103, *p* = .0437, ${\text{η}}_{\text{p}}^{2}$ = 0.013, indicating that duration was significantly longer in the standing condition than in the supine condition when the visual stimuli represented downward self-motion.

The simple main effect of direction of vection on posture was significant in both the standing, *F*(3, 228) = 6.499, *p* < .0003, ${\text{η}}_{\text{p}}^{2}$ = 0.079, and supine, *F*(3, 228) = 4.292, *p* < .0057, ${\text{η}}_{\text{p}}^{2}$ = 0.053, conditions. In the standing condition, multiple comparison analyses (two-tailed *t* tests with a Bonferroni correction) indicated that the mean duration was longer for the downward direction than for the forward direction and for the downward direction than for the backward direction (*p* values < .05/6). In the supine condition, multiple comparison analyses indicated that the mean duration was longer for the upward direction than for the forward direction, for the downward direction than for the forward direction, and the forward direction than for the backward direction (*p* values < .05/6). The other interactions did not reach significance: Age × Posture, *F*(1, 76) = 0.149, *p* = .7009, ${\text{η}}_{\text{p}}^{2}$ = 0.002; Age ×Direction, *F*(3, 228) = 1.511, *p* = .2125, ${\text{η}}_{\text{p}}^{2}$ = 0.019; and Age × Posture × Direction, *F*(3, 228) = 1.481, *p* = .2206, ${\text{η}}_{\text{p}}^{2}$ = 0.019.

#### Magnitude

A three-way mixed ANOVA (2 age × 2 posture × 4 direction conditions) revealed significant main effects of age, *F*(1, 76) = 4.728, *p* = .0328, ${\text{η}}_{\text{p}}^{2}$ = 0.059, and direction, *F*(3, 228) = 7.050, *p* = .0001, ${\text{η}}_{\text{p}}^{2}$ =0.085, whereas the main effect of posture was not significant, *F*(1, 76) = 1.311, *p* = .2558,  = 0.017 (Figure 4C). Multiple comparison analyses (two-tailed *t* tests with a Bonferroni correction) of the main effect of direction indicated that the mean magnitude was greater for upward vection than for forward vection, for downward vection than for forward vection, and for forward vection than for backward vection (*p* values < .05/6). None of the interactions reached significance: Age × Posture, *F*(1, 76) = 0.086, *p* = .7700, ${\text{η}}_{\text{p}}^{2}$ = 0.001; Age × Direction, *F*(3, 228) = 1.703, *p* = .1672, ${\text{η}}_{\text{p}}^{2}$= 0.022; Posture × Direction, *F*(3, 228) = 1.927, *p* = .1261, ${\text{η}}_{\text{p}}^{2}$= 0.025; and Age ×Posture × Direction, *F*(3, 228) = 0.243, *p* = .8663, ${\text{η}}_{\text{p}}^{2}$ = 0.003.

### Discussion

In Experiment 2, vection tended to be more rapid and stronger in children than in adults such that latency and magnitude were significantly shorter and greater, respectively, in children than in adults. This trend is consistent with the findings of previous studies that reported significantly more rapid and stronger vection in school-age children than in adults (Shirai et al., 2012, 2014, 2018).

Experiment 2 also showed that vection seemed to be more rapid and lasted longer in the standing condition than in the supine condition in both children and adults. Although there was no significant main effect of posture in any of the three measurements (latency, duration, and magnitude), the interaction between posture and direction was significant for both latency and duration. The interaction and relevant multiple comparison analyses for the simple main effects of posture on vection direction indicated that reported vection was more rapid and longer lasting in the standing condition than in the supine condition in some vection directions (latency: downward and forward; duration: downward). In conjunction with the lack of any significant interactions relevant to age, these results imply that both the children and the adults had a tendency to experience more rapid and longer lasting vection in the standing condition than in the supine condition.

In addition, multiple comparison analyses of the simple main effect of vection direction on posture for latency and duration revealed that vection seemed to be more rapid and longer lasting in the vertical directions than in the longitudinal directions in the head-centered coordinate for both children and adults. More specifically, latency was significantly shorter in downward vection than in backward vection in both the standing and supine conditions, whereas duration was significantly longer in downward vection than in forward vection and in downward vection than in backward vection in the standing condition and significantly longer in upward vection than in forward vection and in downward vection than in forward vection in the supine condition. Similar results were observed for magnitude. The significant main effect of vection direction and multiple comparison analyses for the main effect revealed that magnitude was significantly greater in upward vection than in forward vection and in downward vection than in forward vection.

Taken together, the results of Experiment 2 indicate that vection seemed to be induced more rapidly and strongly in children than in adults, as shown in previous studies. However, regarding the relationship between an observer’s body and gravitational axes, the characteristics of vection were comparable between the two age groups.

## General Discussion

The primary aim of this study was to investigate the development of the effects of the interaction between the body axis and gravitational axis on vection when viewing visual stimuli. Experiment 1 examined the effects of congruency/incongruency between the body and gravitational axes on vection in adults to determine the appropriate experimental conditions for the subsequent experiment that also included child observers (Experiment 2).

The results of Experiment 1 indicated that adult participants experienced vection more rapidly and strongly when their body axis and the gravitational axis were consistent (standing condition) compared with when their body axis was orthogonal to the gravitational axis (supine condition). Moreover, adult participants showed more rapid and stronger vection in the vertical directions than in the longitudinal directions in the head-centered coordinate, independent of the congruency/incongruency between the orientation of the body and gravitational axes.

Experiment 2 further investigated the effects of the relationship between the body and gravitational axes on vection that were observed in Experiment 1 using both children and adults to assess the development of this relationship. The results of Experiment 2 share similarities with the results of Experiment 1 for both children and adults. Specifically, both children and adults tended to show more rapid and longer lasting vection in the standing condition than in the supine condition and for the vertical directions than for the longitudinal directions in the head-centered coordinate. In addition, as previously reported (Shirai et al., 2012, 2014, 2018), vection was induced more rapidly and strongly in children than in adults. Taken together, the present results imply that the effects of the body and gravitational axes on induced vection were comparable between school-age children and adults but that more rapid and longer lasting vection occurred in children.

In other words, patterns of differences in induced vection among different experimental conditions are similar between school-age children and adults (e.g., the relationship between an observer’s body orientation and gravitational axes), but the entire effect of vection (e.g., latency and magnitude) is very different between these two age groups. These results are consistent with previous findings. For example, Shirai et al. (2018) reported that asymmetry in forward vection and backward vection is commonly observed in both school-age children and adults (vection can be induced more rapidly and strongly by visual stimuli representing backward self-motion than by stimuli representing forward self-motion). However, the authors also observed significantly more rapid and stronger vection in children than in adults. Taken together, the present and previous results imply that the patterns of relative difference in induced vection among different experimental conditions may develop into adult-like forms at a relatively early stage of life, whereas the entire effect of vection may develop more protractedly.

One may claim that the results of Experiment 1 and Experiment 2 seem to be inconsistent because of the lack of the significant main effect of posture in Experiment 2. This inconsistency between Experiments 1 and 2 might stem from the difference in the experimental setting; Experiment 1 contained three speed conditions, whereas Experiment 2 had only one speed condition. Thus, each participant in Experiment 1 had to engage in more experimental trials than the participants in Experiment 2. Such difference(s) in experimental procedure could be critical for producing the inconsistent results between Experiments 1 and 2. However, the results of Experiment 2 indicated significant interaction between posture and direction in both latency and duration, which seems to be partially consistent with the main effect of posture observed in Experiment 1; the interaction showed that both children and adults experienced more rapid and longer lasting vection in the standing condition than in the supine condition in several vection directions (latency: downward and forward; duration: downward). In addition, it should be noted that there were no results showing that vection was more rapid and longer lasting in the supine posture than in the standing posture in Experiment 2. This means that, although the main effect of posture was not significant, the participants of Experiment 2 tended to show more rapid and longer lasting vection in the standing condition than in the supine condition.

This study found that reported vection was more rapid and longer lasting when the body and gravitational axes were congruent than when the body and gravitational axes were orthogonal to each other. It is possible that vection can be induced more rapidly and lasts longer when nonvisual information such as vestibular and proprioceptive inputs and/or the recognition of the observer’s own postural state (e.g., such as standing on the ground vs. lying on the ground) signals that the orientation of the observer’s body is congruent with the gravitational axis. In the present experiments, although the visual stimuli in the two posture conditions (standing and supine) were identical, the relevant nonvisual information, such as vestibular and proprioceptive inputs and/or higher cognitive information about the observer’s own postural state, differed markedly between the two posture conditions. These findings seem to be consistent with several previous studies (e.g., Guterman & Allison, 2019; Guterman et al., 2012; Tovee, 1999).

An ecological viewpoint may explain the current and previous results, showing the advantage of the consistency between body-gravity axes in vection. As discussed by Guterman et al. (2012), the body axis is congruent with the gravitational axis during locomotion such as walking or driving in most cases, and lying postures such as supine posture are less common during locomotion. Thus, it is plausible that any information signaling congruency (or incongruency) between the body and gravitational axes strengthens (or weakens) self-motion perception, such as vection (see also the discussion of Nakamura & Shimojo, 1998).

Alternatively, interpretation of the effects of different postures on induced vection is that the size of the supporting surface between an observer’s body and the ground plane may influence the induction of vection. Seno et al. (2013) reported that physical restriction of an observer’s mobility (e.g., wearing heavy iron clogs) inhibited vection; the authors suggested that the observer’s recognition of difficulty during locomotion inhibited vection. A similar interpretation may be applicable to the present findings. In the supine condition, a relatively large area of each observer’s body (almost all of his/her back, the back of the thighs, buttocks, calves, etc.) was necessarily in direct contact with the ground plane. On the other hand, in the standing condition, a very small portion of each observer’s body (only the soles of the feet) was in direct contact with the ground. The larger size of the contact area between the observer’s body and the ground plane in the supine condition may have resulted in restriction of the observer’s own mobility, as a larger contact area may cause greater friction between the observer’s body and the ground.

## Concluding Remarks

The present study revealed that both school-age children and adults tended to experience more rapid and longer lasting vection when the body and gravitational axes were congruent (standing condition) than when these axes were orthogonal to each other (supine condition). On the other hand, school-age children exhibited significantly more rapid and longer lasting vection than adults, as previously reported (Shirai et al., 2012, 2014, 2018). These results imply that although the quantitative aspects of vection (e.g., latency and magnitude) differed robustly between school-age children and adults, some qualitative aspects of vection (e.g., the relative difference in induced vection among different viewing conditions for visual stimuli) were comparable in children and adults. Further studies assessing the development of the qualitative aspects of vection are required to understand the whole developmental process of self-motion perception in human beings.
